# The complete mitochondrial genome of *Peleteria iavana* (Diptera, Tachinidae) in Guizhou, China, and its phylogenetic placement

**DOI:** 10.1080/23802359.2023.2194457

**Published:** 2023-03-29

**Authors:** Peng Zhang, Yan Zhi, Chunchun Guo, Chuntian Zhang, Jiayu Liu

**Affiliations:** a Key Laboratory of Medical Insects, Guizhou Medical University, Guiyang, P.R. China; b School of Basic Medical Science, Guizhou Medical University, Guiyang, P.R. China; c Laboratory Animal Center, Guizhou Medical University, Guiyang, P.R. China; d College of Life Sciences, Shenyang Normal University, Shenyang, P.R. China

**Keywords:** *Peleteria iavana* (Wiedemann, 1819), mitochondrial genome, phylogeny, Tachinidae

## Abstract

The mitochondrial genome of the tachinid fly *Peleteria iavana* (Wiedemann, 1819), which belongs to the family Tachinidae, was obtained for the first time using high-throughput sequencing techniques. The complete mitochondrial genome is 15,697 bp in size and consists of 13 protein-coding genes (PCGs), two ribosomal RNA genes, 22 transfer RNAs and a non-coding control region. The nucleotide composition biases A and T, the overall A + T percentage is up to 78.9% of the entire mitogenome. A phylogenetic analysis of 30 species within the family Tachinidae suggested that *P. iavana* is most closely related to (*Janthinomyia* sp.+*Lydina aenea*). The *P. iavana* mitochondrial genome will be a fundamental resource for understanding the molecular phylogenetic relationships of the species-rich subfamily Tachininae of Tachinidae.

## Introduction

The Tachininae is the second most speciose subfamily in Tachinidae, with 2,746 described species worldwide (O’Hara et al. 2020). As a parasitoid, it is an important natural enemy in most terrestrial ecological communities (Stireman et al. 2006). Genus *Peleteria* is a representative member of Tachininae, including 4 subgenera and 124 species in the world, while 2 species occur in Guizhou Province, China (O’Hara et al. 2009; 2020). *Peleteria iavana* (Wiedemann, 1819) spreads throughout the Palearctic, Oriental, Afrotropical and Australasian Regions (O’Hara and Henderson 2020), and it is considered a native species of China (O’Hara et al. 2009). In this study, the mitogenome data of *P. iavana* is sequenced and annotated as the first mitogenome of genus *Peleteria*, and the phylogenetic placement of *P. iavana* in the family Tachinidae is explored.

## Materials and methods

**Figure 1.**
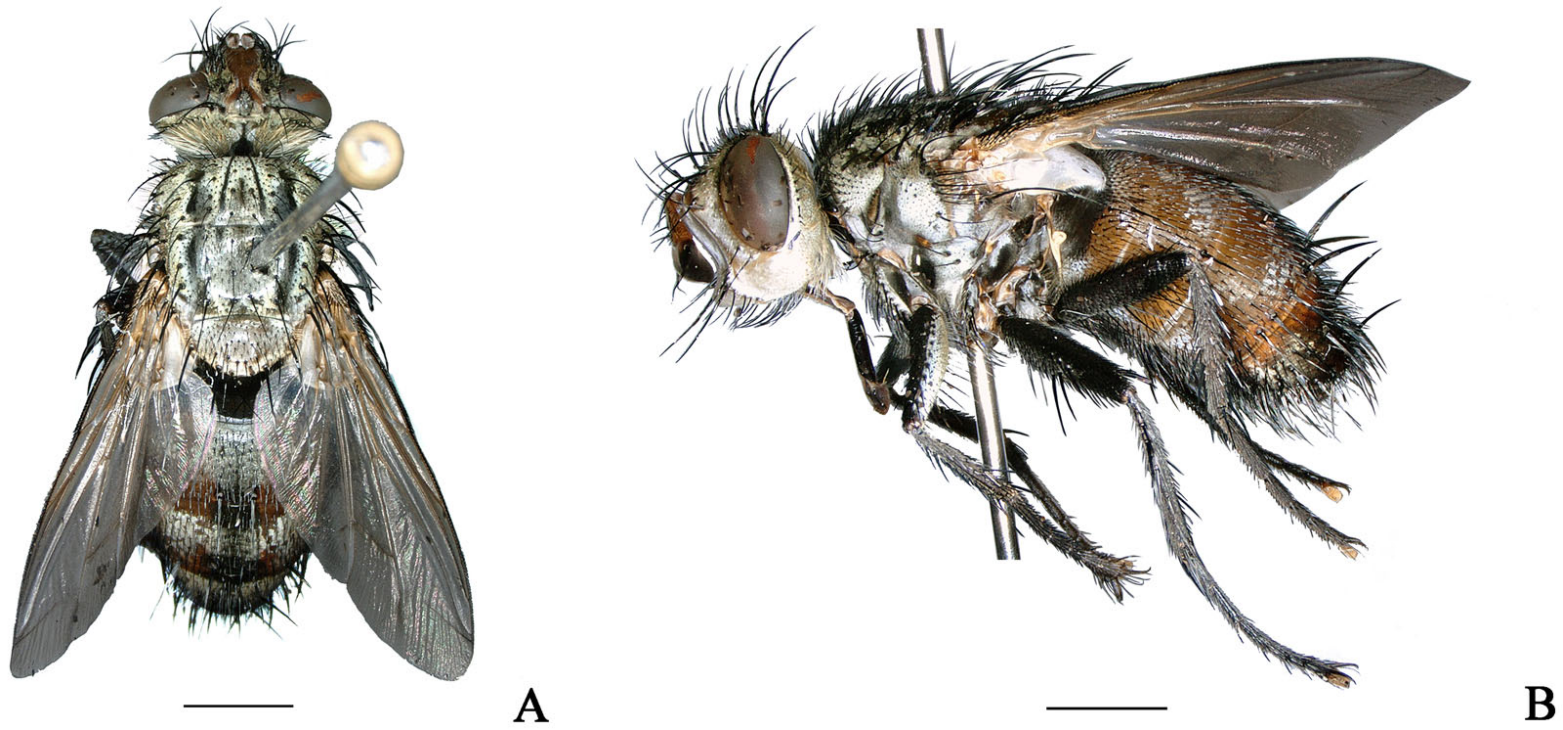
Adult of *Peleteria iavana* (Wiedemann, 1819). (A) Dorsal view; (B) Lateral view. Scale bars: 2.0 mm. (photography by Peng Zhang).

The specimen (Figure 1) was collected by sweeping collection from Beipanjiang Town, Zhenfeng County, Guizhou Province, China (25°31′48″N, 105°45′36″E) on 26 July 2021 and was deposited at the Key Laboratory of Medical Insects of Guizhou Medical University (https://www.gmc.edu.cn/, Jiayu Liu, fsliujiayu@163.com) under the voucher number PI20210726. Photographs were taken with the KEYENCE VHX-6000 system. The sequencing library was established and then used the Illumina Hiseq PE150 platform for whole genome sequencing. The raw reads were assembled using Mitoz 2.4-alpha with default parameters (Meng et al. 2019). The assembled contigs were aligned with the previously published mitochondrial genomes of *Hamaxiella brunnescens* (MW256712.1) and *Janthinomyia* sp. (MK644822.1) using Geneious Prime 2020.2.2 (Kearse et al. 2012). Annotation of the mitogenome, including gene prediction and non-coding RNA analysis, was conducted using MITOS2 Web Server (http://mitos2.bioinf.uni-leipzig.de/index.py) (Bernt et al. 2013) and tRNAscan-SE version 1.21 (Schattner et al. 2005). Subsequently, the mitogenome data were submitted to GenBank database through NCBI.

To reveal the phylogenetic position of *P. iavana*, the dataset of 13 PCGs of previously reported 29 tachinid species were downloaded and their GenBank accession numbers were provided in Figure 3 (Nelson et al. 2012; Shao et al. 2012; Zhao et al. 2013; Li et al. 2017; Hou et al. 2018, 2019; Pei et al. 2019; Seo et al. 2019; Luo et al. 2021; Yan et al. 2021; Li et al. 2022; Wang, Zhi, Zhang et al. 2022; Wang, Zhi, Yao et al. 2022). Additionally, *Fucellia costalis* from Anthomyiidae and *Sarcophaga ruficornis* from Sarcophagidae were chosen as outgroups. Preliminary multiple sequence alignments were performed using the Clustal W in MEGAX 10.2.6 (Kumar et al. 2018). The phylogenetic tree was reconstructed in the maximum-likelihood (ML) method with 1,000 bootstrap repeats using MEGA X 10.2.6 (Kumar et al. 2018).

## Results

**Figure 2.**
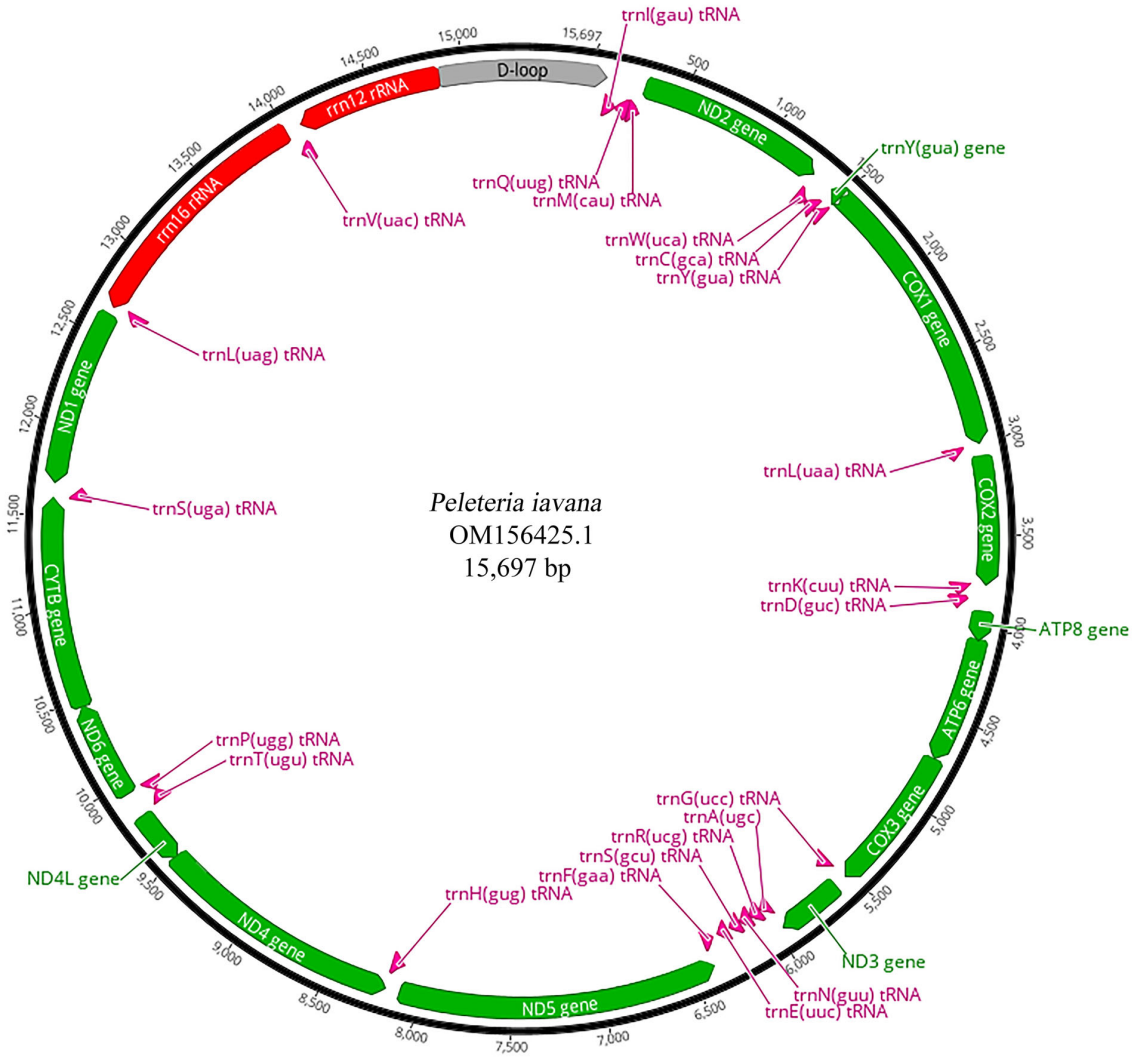
The mitochondrial genome map of *Peleteria iavana*. Arrangement of 37 genes represented in the map, including 13 protein-coding genes, 22 tRNA genes, and two rRNA genes. A circular mitochondrial genome map was drawn using Geneious Prime 2020.2.2 (Kearse et al. 2012). 
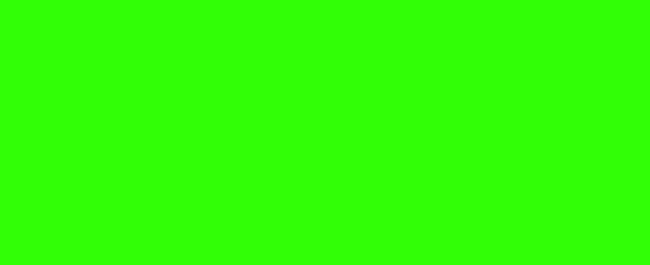
 protein-coding gene, 
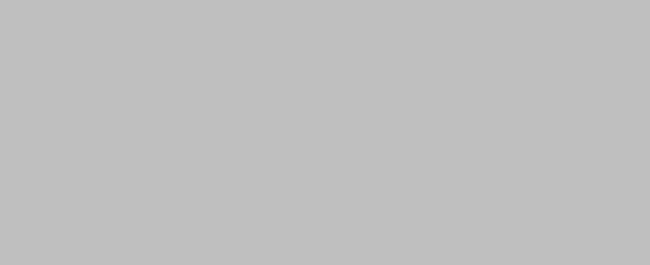
 control region, 
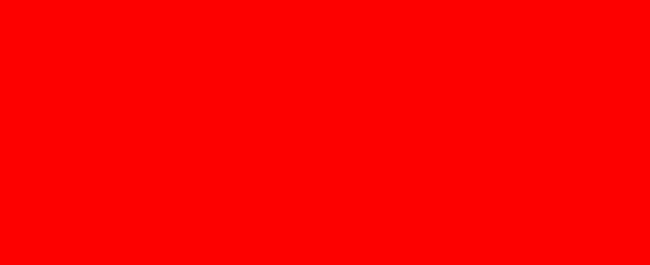
 rRNA gene, 
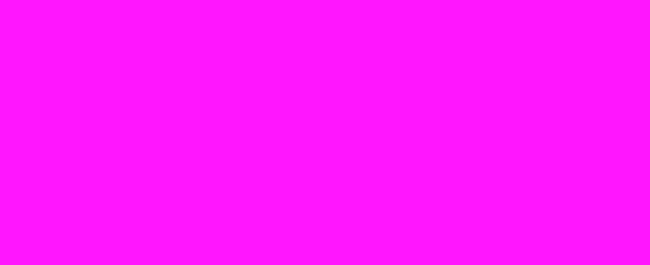
 tRNA gene.

The 4.43 Gb raw data was yielded by the high-throughput sequencing techniques with 12,926,828 reads, and the 4.29 Gb (96.84%) clean data was obtained after filtering. The complete mitochondrial genome of *P. iavana* was assembled using 594,117 reads, and the average depth of coverage was assessed at 4561.6 X (Supplementary Figure 1). The average coverage depth for two regions nucleotides 14,853–14,977 (A + T ratio: 94.4%) and nucleotides 15,147–15,390 (A + T ratio: 93.5%) was 503.1 X (max: 635 X, min: 426 X) and 306.8 X (max: 632 X, min: 138 X), respectively. The reason that the two regions had relatively low coverage depth in the mitochondrial genome may be that the control region had a higher AT ratio and more repeat sequences. The complete circular mitogenome of *P. iavana* (GenBank accession number: OM156425.1) was 15,697 bp in length, with the base composition as follows: A (40.5%), T (38.4%), G (9.0%), and C (12.1%). The nucleotide composition was AT-biased (A + T ratio: 78.9%). It contained 13 protein-coding genes (PCGs), two rRNA genes, 22 tRNA genes and one non-coding control region, which was a typical configuration of the tachinid mitogenome (Figure 2). Four PCGs, two rRNA genes and eight tRNA genes were encoded by the light strand, while others were located on the heavy strand.

The 13 PCGs accounted for 71.1% of the complete mitogenome of *P. iavana* (11,160 bp). PCGs utilized a variety of start codons including the standard ATN, except for the nonstandard TCG (*COI*) and TTG (*NDI*). The most frequent start codon was ATG, which covered six PCGs (*COII*, *ATP6*, *COIII*, *ND4*, *ND4L*, *CYTB*). Five PCGs (*ND2*, *ATP8*, *ND3*, *ND5*, *ND6*) started with ATT codon. The stop codon TAA was assigned to most of the PCGs (*ND2*, *ATP8*, *ATP6*, *COIII*, *ND3*, *ND4L*, *ND6* and *ND1*), but an incomplete stop codon T was used by four PCGs (*COI*, *COII*, *ND5* and *ND4*), *CYTB* terminated with stop codon TAG. Besides, the length of 22 tRNAs in the mitogenome ranged from 63 bp (*tRNA^Ala^*, *tRNA^Arg^*, *tRNA^Cys^*) to 72 bp (*tRNA^Val^*).

**Figure 3.**
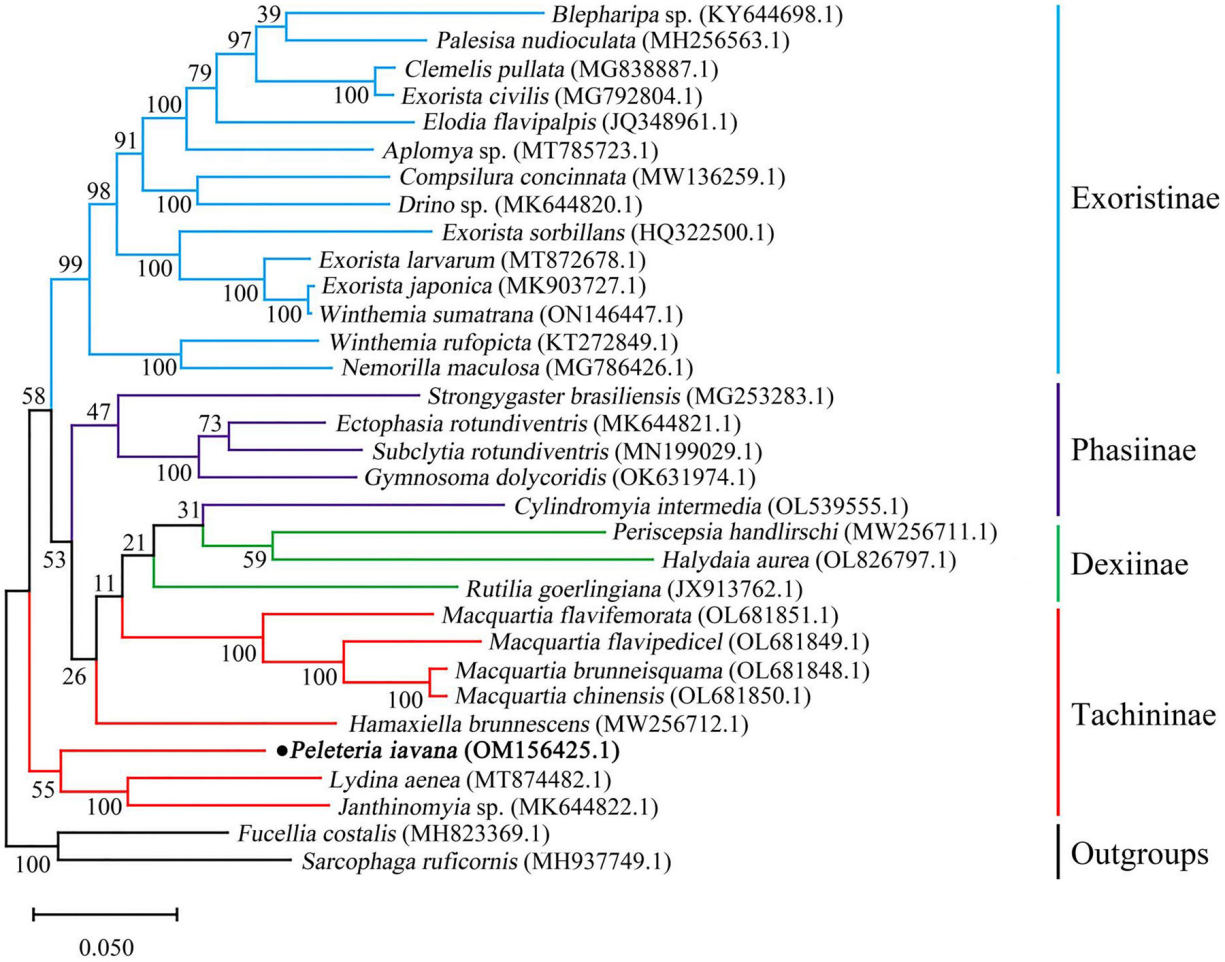
The maximum-likelihood phylogeny of 30 tachinid species from analysis of the combined 13 protein-coding genes dataset. The bolded scientific name indicated this study.

As shown in Figure 3, a phylogenetic analysis based on 13 PCGs revealed that *P. iavana* and (*Janthinomyia* sp.+*Lydina aenea*) clustered together. Additionally, *Hamaxiella brunnescens is* closely related to ((*Cylindromyia*+Dexiinae)+*Macquartia* spp.). Therefore, the subfamily Tachininae was not assigned to be a monophyletic group.

## Conclusion

In the present study, we first reported the mitochondrial genome of *P. iavana.* It was a closed circular molecule of 15,697 bp in length, containing 13 protein-coding genes (PCGs), 22 transfer RNA (tRNA) genes, two ribosomal RNA (rRNA) genes, and a control region. The phylogenetic analysis suggested that Tachinidae was a monophyletic group and subfamily Tachininae has not formed a monophyly. *P. iavana* and (*Janthinomyia* sp.+*Lydina aenea*) clustered together in the basal position. *Hamaxiella brunnescens* of tribe Palpostomatini from subfamily Tachininae was the sister group of (Dexiinae + Cylindromyini), the result was consistent with the previous study on the phylogeny of Tachinidae based on the morphology and molecular data (Stireman et al. 2019). Eventually, as the sister group to *Hamaxiella brunnescens*, four species of genus *Macquartia* clustered together with high supporting values, the phylogenetic tree showed a similarity to the phylogenetic relationships of the Tachinidae in Li et al. (Li et al. 2022). These results provided an important basis for further studies on the mitochondrial genomics and phylogenetics of the family Tachinidae.

## Ethical approval

Ethical approval (No. 1900074) was granted by the Animal Care Welfare Committee of Guizhou Medical University for the study.

## Supplementary Material

Supplemental Material

Supplemental Material

Supplemental Material

Supplemental Material

## Data Availability

The genome sequence data that support the findings of this study are openly available in GenBank of NCBI at https://www.ncbi.nlm.nih.gov/ under the accession no. OM156425.1. The associated BioProject, SRA and Bio-Sample numbers are PRJNA800680, SRR18030147 and SAMN25276555, respectively.

## References

[CIT0001] Bernt M, Donath A, Jühling F, Externbrink F, Florentz C, Fritzsch G, Pütz J, Middendorf M, Stadler PF. 2013. MITOS: improved *de novo* metazoan mitochondrial genome annotation. Mol Phylogenet Evol. 69(2):313–319. 22982435 10.1016/j.ympev.2012.08.023

[CIT0002] Hou P, Ding SM, Li X, Yang D, Zhang CT, Wang Q. 2018. The mitochondrial genome of *Drino* sp. (Diptera, Tachinidae). Mitochondrial DNA B Resour. 3(2):886–887. 33474354 10.1080/23802359.2018.1501318 PMC7799928

[CIT0003] Hou P, Gao S, Li X, Yang D, Zhang CT. 2019. The mitochondrial genome of *Janthinomyia* sp. (Diptera, Tachinidae). Mitochondrial DNA B Resour. 4(1):1601–1602.

[CIT0004] Kearse M, Moir R, Wilson A, Stones-Havas S, Cheung M, Sturrock S, Buxton S, Cooper A, Markowitz S, Duran C, et al. 2012. Geneious basic: an integrated and extendable desktop software platform for the organization and analysis of sequence data. Bioinformatics. 28(12):1647–1649. 22543367 10.1093/bioinformatics/bts199 PMC3371832

[CIT0005] Kumar S, Stecher G, Li M, Knyaz C, Tamura K. 2018. MEGA X: molecular Evolutionary Genetics Analysis across computing platforms. Mol Biol Evol. 35(6):1547–1549. 29722887 10.1093/molbev/msy096 PMC5967553

[CIT0006] Li HN, Zhang BH, Pei WY, Sun HR, Chen JL, Gao XZ, Peng HL, Zhang D, Zhang CT. 2022. Four new species of *Macquartia* (Diptera: Oestroidea) from China and phylogenetic implications of Tachinidae. Insects. 13(12):1096. 36555006 10.3390/insects13121096 PMC9781235

[CIT0007] Li X, Ding SM, Hou P, Liu XY, Zhang CT, Yang D. 2017. Mitochondrial genome analysis of *Ectophasia rotundiventris* (Diptera, Tachinidae). Mitochondrial DNA B Resour. 2(2):457–458. 33490457 10.1080/23802359.2017.1357447 PMC7800338

[CIT0008] Luo Y, Zhi Y, Zhang CT, Yang M, Liu JY. 2021. Complete mitochondrial genome of *Compsilura concinnata* (Meigen) (Diptera, Tachinidae). Mitochondrial DNA B Resour. 6(3):905–906. 33796675 10.1080/23802359.2021.1886020 PMC7995883

[CIT0009] Meng G, Li Y, Yang C, Liu S. 2019. MitoZ: a toolkit for animal mitochondrial genome assembly, annotation and visualization. Nucleic Acids Res. 47(11):e63–e63. 30864657 10.1093/nar/gkz173 PMC6582343

[CIT0010] Nelson LA, Lambkin CL, Batterham P, Wallman JF, Dowton M, Whiting MF, Yeates DK, Cameron SL. 2012. Beyond barcoding: a mitochondrial genomics approach to molecular phylogenetics and diagnostics of blowflies (Diptera: calliphoridae). Gene. 511(2):131–142. 23043935 10.1016/j.gene.2012.09.103

[CIT0011] O’Hara JE, Henderson SJ. 2020. World genera of the Tachinidae (Diptera) and their regional occurrence. Version 11.0. PDF document, pp. 90. https://www.uoguelph.ca/nadsfly/Tach/WorldTachs/Genera/Gentach_ver11.pdf

[CIT0012] O’Hara JE, Henderson SJ, Wood DM. 2020. Preliminary checklist of the Tachinidae (Diptera) of the world. Version 2.1. PDF document, pp. 1039. https://www.uoguelph.ca/nadsfly/Tach/WorldTachs/Checklist/Tachchlist_ver2.1.pdf

[CIT0013] O’Hara JE, Shima H, Zhang CT. 2009. Annotated catalogue of the Tachinidae (Insecta: diptera) of China. Zootaxa. 2190(1):1–236.

[CIT0014] Pei WY, Yan LP, Yang N, Zhang CT, Zheng CY, Yang J, Zhang D. 2019. First report of mitogenome of *Subclytia rotundiventris* (Diptera, Tachinidae) yielded by next-generation sequencing. Mitochondrial DNA B Resour. 4(2):2910–2911. 33365787 10.1080/23802359.2019.1661297 PMC7707014

[CIT0015] Schattner P, Brooks AN, Lowe TM. 2005. The tRNAscan-SE, snoscan and snoGPS web servers for the detection of tRNAs and snoRNAs. Nucleic Acids Res. 33(Web Server):W686–W689. 15980563 10.1093/nar/gki366 PMC1160127

[CIT0016] Seo BY, Cho J, Lee GS, Park J, Park J. 2019. The complete mitochondrial genome of *Exorista japonica* (Townsend, 1909) (Diptera: tachinidae). Mitochondrial DNA B Resour. 4(2):2244–2245. 33365494 10.1080/23802359.2019.1624648 PMC7687561

[CIT0017] Shao YJ, Hu XQ, Peng GD, Wang RX, Gao RN, Lin C, Shen WD, Li R, Li B. 2012. Structure and evolution of the mitochondrial genome of *Exorista sorbillans*: the Tachinidae (Diptera: calyptratae) perspective. Mol Biol Rep. 39(12):11023–11030. 23053992 10.1007/s11033-012-2005-1

[CIT0018] Stireman JO, O'Hara JE, Wood DM. 2006. Tachinidae: evolution, behavior, and ecology. Annu Rev Entomol. 51:525–555. 16332222 10.1146/annurev.ento.51.110104.151133

[CIT0019] Stireman JO, Cerretti P, O'Hara JE, Blaschke JD, Moulton JK. 2019. Molecular phylogeny and evolution of world Tachinidae (Diptera). Mol Phylogenet Evol. 139:106358. 30584917 10.1016/j.ympev.2018.12.002

[CIT0020] Wang R, Zhi Y, Zhang CT, Yang M, Liu JY. 2022. Complete mitochondrial genome of *Cylindromyia* (*Calocyptera*) *intermedia* from Guiyang, China, and phylogeny of Phasiinae (Diptera: tachinidae). Mitochondrial DNA B Resour. 7(6):1008–1010. 35756432 10.1080/23802359.2022.2080021 PMC9225719

[CIT0021] Wang R, Zhi Y, Yao QY, Zhang CT, Liu JY. 2022. Characterization of the complete mitochondrial genome of *Gymnosoma dolycoridis* (Diptera, Tachinidae) and phylogenetic analysis. Mitochondrial DNA B Resour. 7(8):1435–1437. 35958060 10.1080/23802359.2022.2107449 PMC9359177

[CIT0022] Yan LP, Pei WY, Zhang CT, Zhang D. 2021. First report of the mitogenome of *Hamaxiella brunnescens* (Diptera, Tachinidae) from Beijing, China. Mitochondrial DNA B Resour. 6(3):862–864. 33796659 10.1080/23802359.2021.1885321 PMC7971339

[CIT0023] Zhao Z, Su TJ, Chesters D, Wang SD, Ho SYW, Zhu CD, Chen XL, Zhang CT. 2013. The mitochondrial genome of *Elodia flavipalpis* Aldrich (Diptera: Tachinidae) and the evolutionary timescale of tachinid flies. PLoS One. 8(4):e61814. 23626734 10.1371/journal.pone.0061814 PMC3634017

