# Supplementary material for: The complete mitochondrial genome of Peleteria iavana (Diptera, Tachinidae) in Guizhou, China, and its phylogenetic placement
Source: Mitochondrial DNA B Resour. 2023 Mar 29;8(3):466–70. doi: 10.1080/23802359.2023.2194457 (PMC10062221; doi:10.1080/23802359.2023.2194457)
Supplement: Supplemental Material [file TMDN_A_2194457_SM4165.docx]

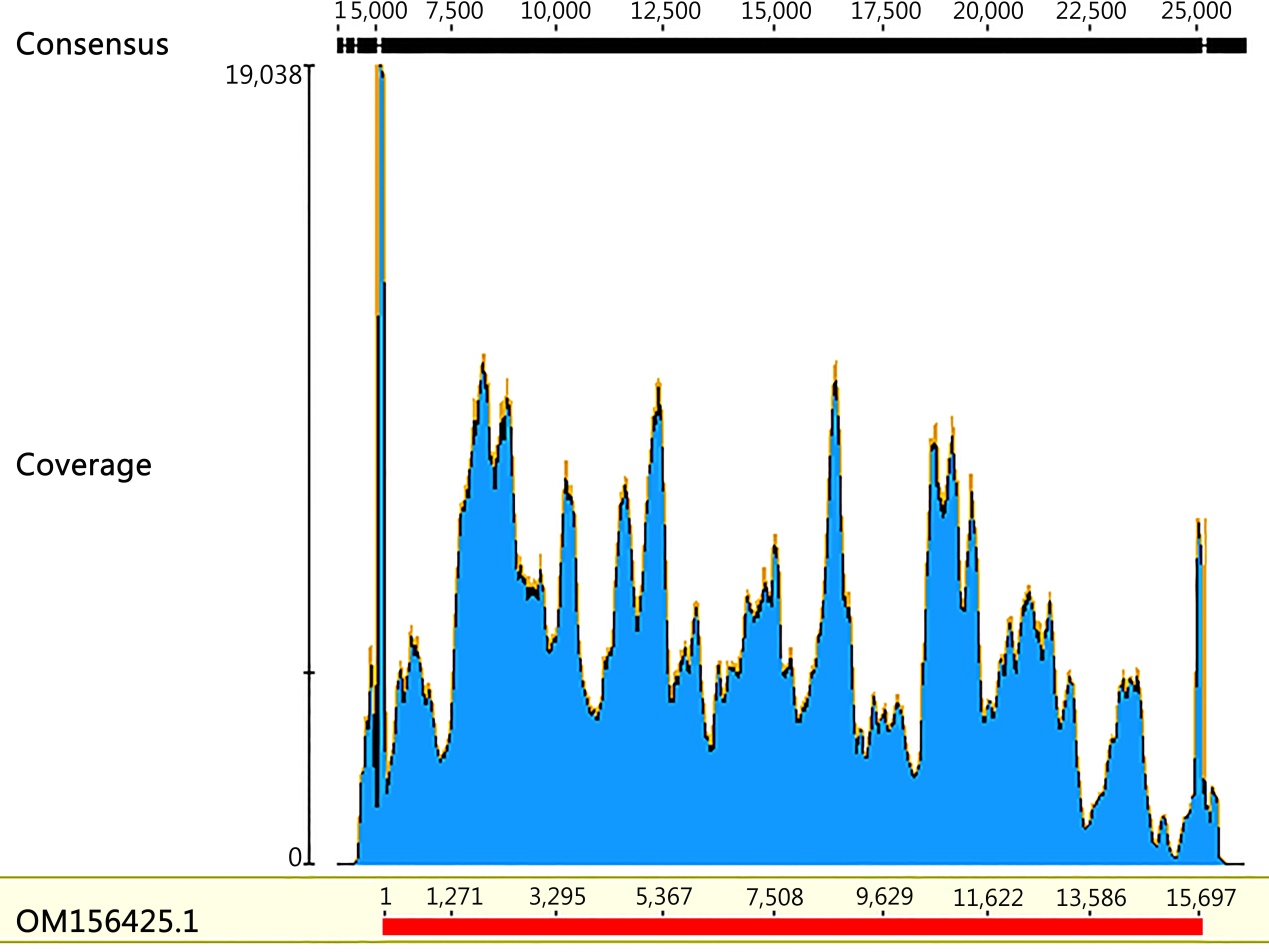


**Supplementary figure 1.** **The coverage depth of mitochondrial genome of *Peleteria iavana*.** ▅ mitochondrial DNA sequence of *Peleteria iavana*, ▅ consensus sequence, ▅ the coverage depth of mitochondrial genome.
